# Basement Membrane Defects in Genetic Kidney Diseases

**DOI:** 10.3389/fped.2018.00011

**Published:** 2018-01-29

**Authors:** Christine Chew, Rachel Lennon

**Affiliations:** ^1^Faculty of Biology Medicine and Health, Wellcome Trust Centre for Cell-Matrix Research, Division of Cell Matrix Biology, School of Biological Sciences, University of Manchester, Manchester, United Kingdom; ^2^Department of Paediatric Nephrology, Royal Manchester Children’s Hospital, Central Manchester University Hospitals NHS Foundation Trust, Manchester Academic Health Science Centre, Manchester, United Kingdom

**Keywords:** basement membrane, glomerulus, Alport syndrome, genetic variation, collagen IV, Pierson syndrome

## Abstract

The glomerular basement membrane (GBM) is a specialized structure with a significant role in maintaining the glomerular filtration barrier. This GBM is formed from the fusion of two basement membranes during development and its function in the filtration barrier is achieved by key extracellular matrix components including type IV collagen, laminins, nidogens, and heparan sulfate proteoglycans. The characteristics of specific matrix isoforms such as laminin-521 (α5β2γ1) and the α3α4α5 chain of type IV collagen are essential for the formation of a mature GBM and the restricted tissue distribution of these isoforms makes the GBM a unique structure. Detailed investigation of the GBM has been driven by the identification of inherited abnormalities in matrix proteins and the need to understand pathogenic mechanisms causing severe glomerular disease. A well-described hereditary GBM disease is Alport syndrome, associated with a progressive glomerular disease, hearing loss, and lens defects due to mutations in the genes *COL4A3, COL4A4*, or *COL4A5*. Other proteins associated with inherited diseases of the GBM include laminin β2 in Pierson syndrome and *LMX1B* in nail patella syndrome. The knowledge of these genetic mutations associated with GBM defects has enhanced our understanding of cell–matrix signaling pathways affected in glomerular disease. This review will address current knowledge of GBM-associated abnormalities and related signaling pathways, as well as discussing the advances toward disease-targeted therapies for patients with glomerular disease.

## Introduction

**Figure 1:**
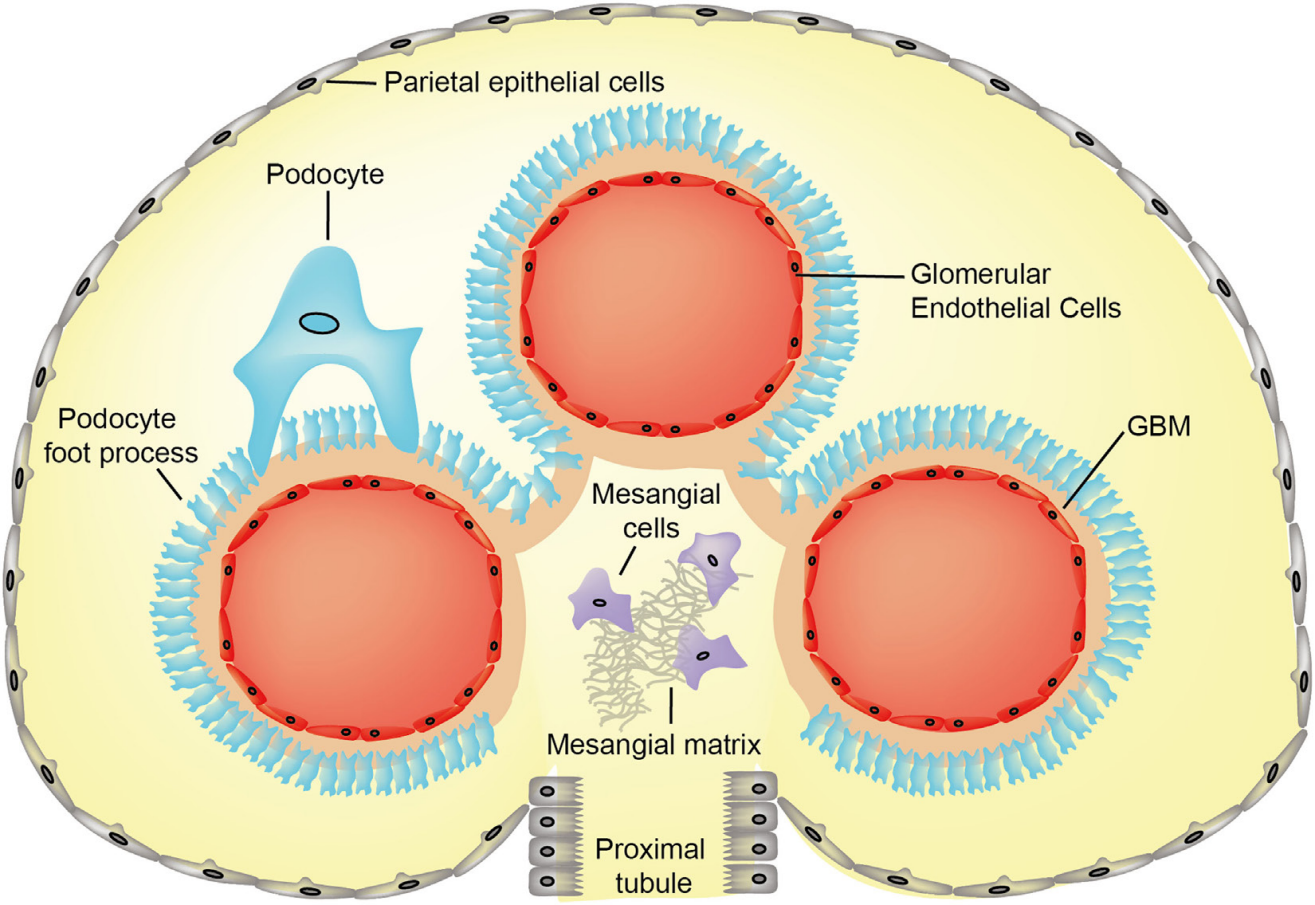
Cross-section image of normal glomerulus showing cellular and extracellular matrix compartments. Abbreviation: GBM, glomerular basement membrane.

The glomerular basement membrane (GBM) is an integral component of the glomerular filtration barrier; an important and highly complex capillary wall that is exposed to mechanical forces driven by capillary hydrostatic pressure. This barrier is permeable to water and small molecules, and selectively withholds cells and macromolecules such as albumin in the circulation (1). During glomerular development, the assembly of endothelial and podocyte layers generates the filtration barrier (2). The GBM separates endothelial cells and podocytes, and it represents a specialized extracellular matrix (ECM), which maintains barrier function (Figure 1). The GBM is formed during glomerulogenesis and is maintained by secreted components from both podocytes and endothelial cells (3–5). The mature, human GBM is relatively thick in comparison to other basement membranes and measures between 300 and 350 nm (4, 6). In addition to the cells of the filtration barrier, mesangial cells (between adjacent capillary loops) and the parietal epithelial cells of Bowman’s capsule are necessary for maintaining intact glomerular function.

The fusion of podocyte and endothelial basement membranes during the development of the GBM creates an intricate meshwork containing laminins, type IV collagen, nidogen, and heparan sulfate proteoglycans (HSPGs) (7–10). Our recent mass spectrometry-based proteomic analysis of human glomerular ECM *in vivo* revealed a more complex composition of 144 structural and regulatory matrix proteins supporting the unique organelle structure of the glomerulus (11). Not surprisingly collagen (types I, IV, and VI), and laminin isoforms were identified as the most abundant components (11). The secretion of matrix molecules into the GBM is likely to be facilitated by cross talk between podocytes and endothelial cells. Indeed, the proteomic investigation of cell-derived ECM isolated from glomerular cells in culture identified 35 highly connected matrix components and a number of these were differentially expressed in mono- versus coculture ECMs (12). Although a unique ECM niche, the GBM contains proteins that are found in other basement membranes; however, the specific arrangement of matrix isoforms in the GBM provides its distinctive composition and function.

**Figure 2:**
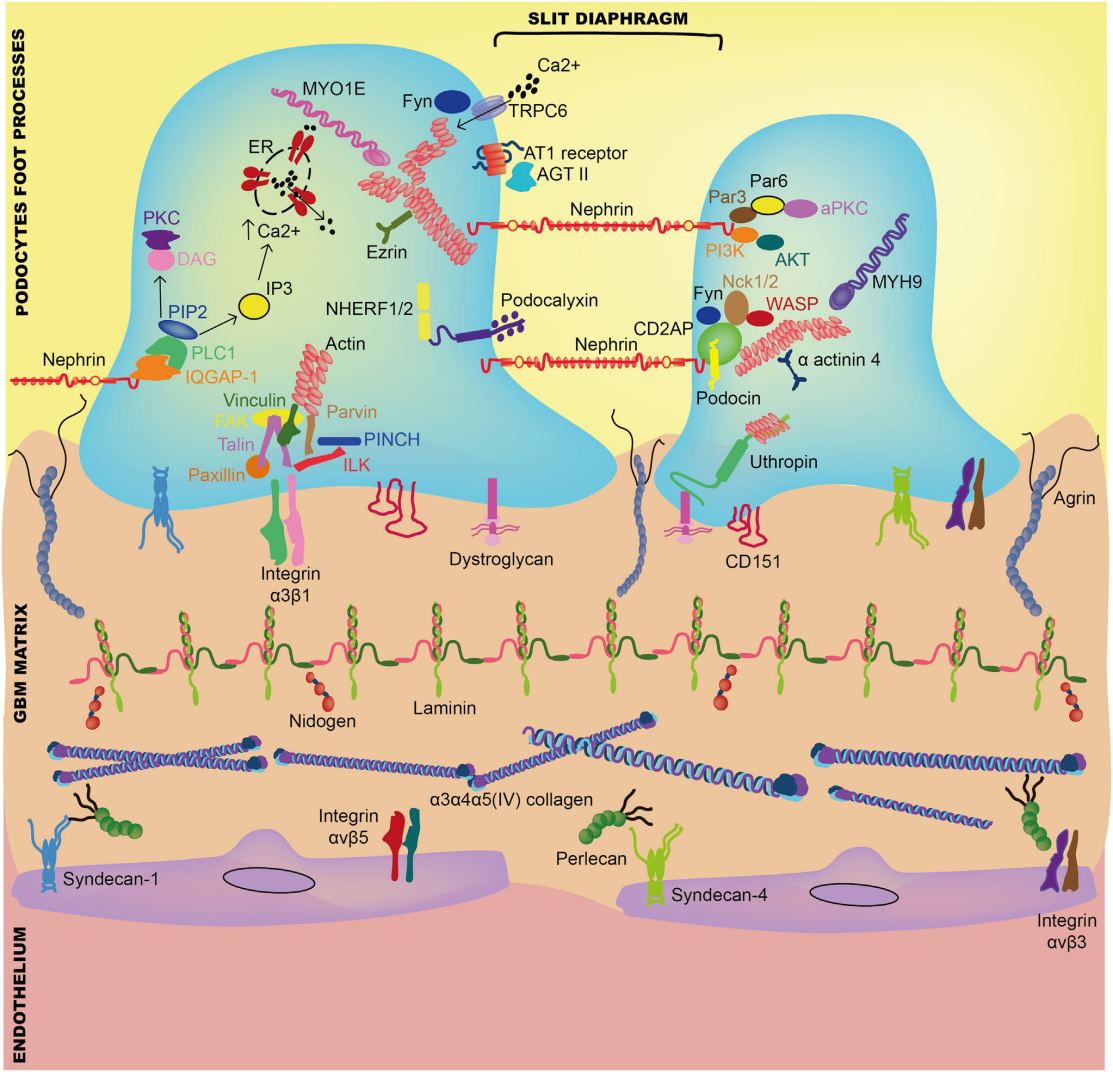
Interaction between components of the glomerular basement membrane and adjacent glomerular cells. Abbreviations: AGT II, angiotensin-II; AKT, protein kinase B; aPKC, atypical protein kinase C; AT1, angiotensin-I; CD2AP, CD2-associated protein; DAG, diacylglycerol; ER, endoplasmic reticulum; FAK, focal adhesion kinase; IQGAP-1, IQ motif containing GTPase-activating protein-1; ILK, integrin-linked kinase; IP3, inositol triphosphate; MYH9, myosin heavy chain 9; MYO1E, myosin-1E; PIP3, phosphatidylinositol 4,5-biphosphate; Nck1, non-catalytic region of tyrosine kinase adaptor protein-1; NHERF1/2, Na^+^/H^+^ exchanger regulatory factor-1/2; PI3K, phosphoinositide 3-kinase; PINCH, Cys-His-rich protein; PKC, protein kinase C; PLC, phosphatidylinositol phospholipase C; TRPC6, transient receptor potential cation channel-6; WASP, Wiskott–Aldrich syndrome protein.

Adhesion receptors such as integrins, syndecans, and dystroglycan connect cells to their associated ECM ligands in the extracellular space and to the cellular cytoskeleton inside the cell (Figure 2). The mature focal adhesion complexes that result from this cell–matrix interaction are vital in all aspects of normal cell development including growth, proliferation, signaling, differentiation, migration, and survival (13, 14). Furthermore, in addition to maintaining GBM structure, secreted growth factors support the ECM through organized cell–cell signaling (13).

The study of ECM components that maintain the integrity of the GBM has advanced the understanding of what constitutes a healthy glomerulus. However, disruption to this specialized ECM niche can alter the function of the filtration barrier and cause the leakage of albumin into the urine (albuminuria). Of the nine major proteins discovered in the GBM, genetic mutations in type IV collagen and laminin are reported to cause glomerular disease in humans (15, 16). Although these distinct genetic mutations have been defined, the pathogenesis of the majority of kidney diseases such as diabetic nephropathy are less clear, and it is thought that environmental influences may have a role. Not all conditions that present with proteinuria and glomerular disease have a genetic component, which is one of the main limitations in the diagnosis and treatment of these rare diseases. Animal models have been beneficial in deciphering pathogenic pathways of disease; however, targeted treatments for genetic diseases of the GBM currently do not exist. This review will cover key findings and recent discoveries of mechanisms that sustain a healthy GBM and known pathogenic pathways that lead to genetic kidney disease. In addition, recent advances and novel approaches in the field of ECM in glomerular health and disease will be discussed.

## GBM Biology in Health and Disease

### Type IV Collagen

**Figure 3:**
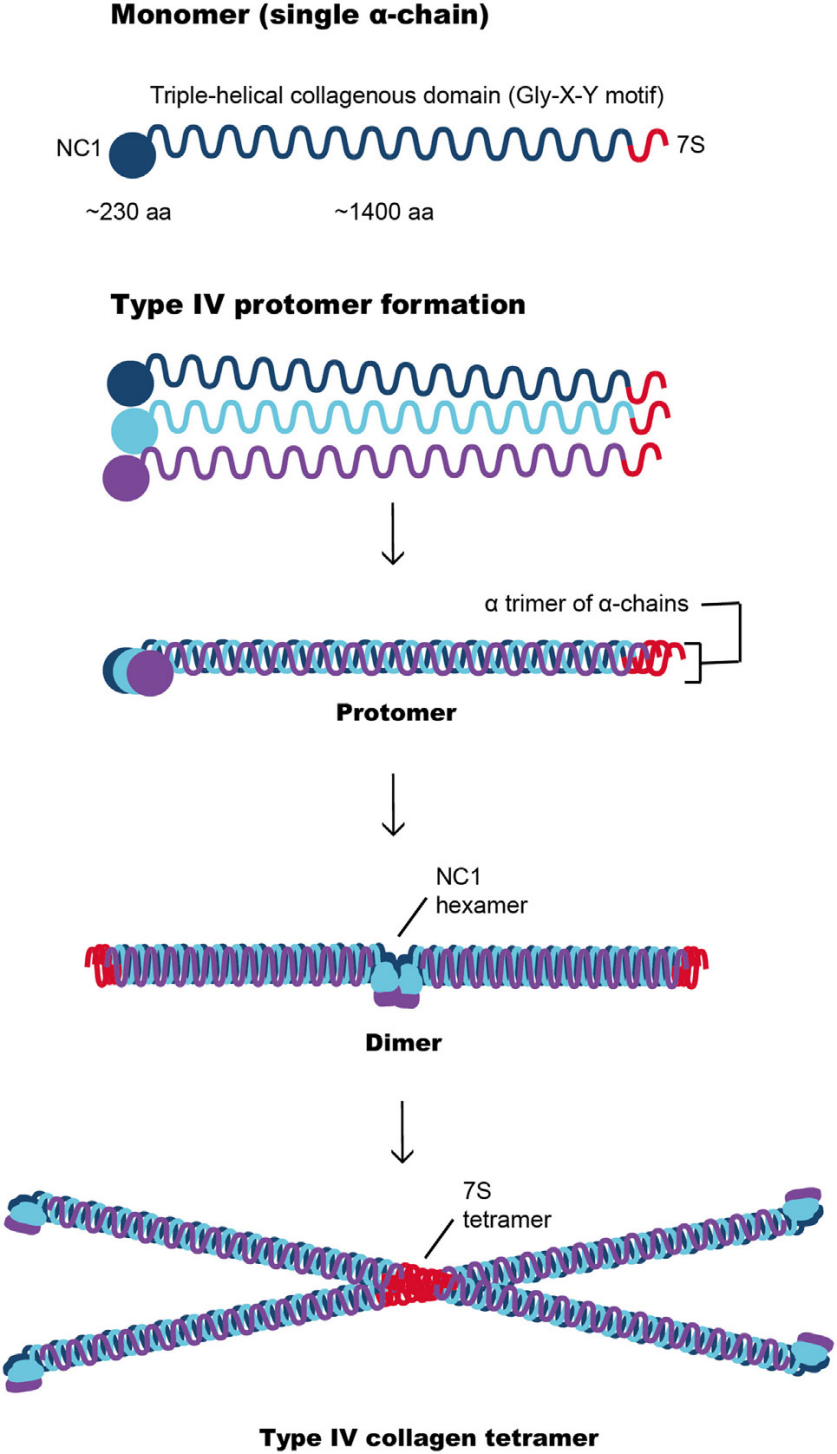
Type IV collagen network formation involving interaction between the α-chains through their NC1 domains to form a dimer or through their 7S domains to produce a tetramer. These complex interactions are important for the development of the type IV collagen scaffold, which are subsequently reinforced by suprastructural associations of collagenous domains.

In common with other basement membranes, type IV collagen forms a major structural component of the GBM and contributes significantly to its stability and assembly (17, 18). There are six collagen IV α-chains, α1(IV) to α6(IV), encoded by the genes *COLA4A1* and *COL4A2* on chromosome 13; *COL4A3* and *COL4A4* on chromosome 2, and *COL4A5* and *COL4A6* on the X chromosome. Each collagen IV α-chain is composed of an N-terminal 7S domain, Gly-X-Y repeats and a non-collagenous (NC1) domain at the C-terminus (Figure 3). It is the unique interrupted Gly-X-Y amino acid triplet repeats within these three domains that give collagen IV its flexible quality, enabling it to successfully form basement membranes (19). The α-chains assemble in the endoplasmic reticulum forming the collagen IV triple helix or protomers, which are released into the extracellular space where they undergo polymerization *via* head-to-head NH2- and COOH-terminal domains that result in unique collagen IV hexameric networks (20).

The six collagen IV α-chains form three distinct networks comprising helical heterotrimers these are α1α1α2(IV), α3α4α5(IV), and α5α5α6(IV), and they are present in different glomerular compartments (20, 21). During glomerulogenesis, there is a shift in the composition of collagen IV α-chains involving a transition from α1α1α2(IV), predominantly found in the immature S-shaped and early capillary loop GBM, to the α3α4α5(IV) network, which defines the mature GBM (22). The α3α4α5(IV) isoform is primarily produced by podocytes, whereas the α1α1α2(IV) is present in all cell types including endothelial cells, podocytes, and mesangial cells (5). Type IV collagen networks differ between species and their expression is not solely restricted to the GBM, but these protomers have also been found in the mesangial matrix and Bowman’s capsule (23, 24). Mechanisms underlying the transition between collagen IV isoforms during glomerular development are not fully understood. However, it is considered that the α3α4α5(IV) is less susceptible to proteolytic degradation than the α1α1α2(IV) isoform, which suggests that it may have a crucial role in maintaining the structural integrity of the GBM (25). The lateral links between the α3α4α5(IV) chains are also essential in fortifying networks within the GBM (26). The α1α1α2(IV) and α3α4α5(IV) networks are both present in the glomerular capillary tuft, whereas the α5α5α6(IV) is localized to the basement membrane of Bowman’s capsule. Overall, the role of type IV collagen networks in supporting the GBM are crucial, and the absence or reduction of the α3α4α5(IV) chains reduces the quantity of protomers secreted, leading to thin basement membranes and Alport syndrome (AS) (27, 28).

#### Alport Syndrome

##### Clinical Features

A diagnosis of AS should be suspected in individuals who present with glomerular hematuria and a family history of AS or renal failure without other identified causes and these individuals should undergo further investigations (29). The diagnosis of Alport was previously confirmed by the presence of lamellated GBM or abnormal deposition of α3α4α5(IV) and α5(IV) collagen chains from the GBM and skin, respectively, the latter of which reported moderate sensitivity in 80% males and high specificity (30). More recently, genetic testing has been a preferred diagnostic method, as it is less invasive than skin or kidney biopsy and has a diagnostic specificity of 95% (29). Sanger sequencing was the gold standard for the diagnosis of AS where other techniques were unsuccessful; however, next-generation sequencing to screen genes corresponding to α3, α4, and α5 chains of type IV collagen has now succeeded this method (31).

The clinical phenotype in AS also includes ocular abnormalities and sensorineural hearing loss. Hearing loss was more likely to occur in 60% of patients with missense mutations before 30 years of age, which is lower than the 90% risk in individuals with other mutations. In the majority of patients with AS, the auditory deficit affects the higher sound frequencies, and it usually progresses to hearing loss within conversational range. The ocular defects in X-linked and autosomal recessive AS include anterior lenticonus, perimacular retinal flecks, posterior corneal vesicles, and recurrent corneal erosions (32).

##### Etiology and Pathogenesis

The significant role of type IV collagen in the maintaining the GBM has been highlighted by mutations in the α3, α4, and α5 chains of type IV collagen encoded by *COL4A3, COL4A4*, and *COL4A5* genes, respectively, which cause AS (15, 33). There are two main modes of inheritance in AS: X-linked and autosomal recessive (30, 34, 35). The X-linked form of AS occurs in around 80% of patients and involves mutations in the *COL4A5* gene encoding the α5 chain of type IV collagen. All affected males with the X-linked AS will ultimately develop end-stage renal disease (ESRD) requiring dialysis or kidney transplantation by at least 60 years of age (30). Female patients with X-linked AS also develop proteinuria; however, only around 30% develop ESRD at 60 years (36). Approximately 15% of patients with AS have the autosomal recessive form of disease arising from mutations in *COL4A3* or *COL4A4*, and the phenotype of these patients is similar to those with X-linked AS. A final small group of patients have heterozygous mutations in *COL4A3* or *COL4A4* and manifest with an AS phenotype.

Genetic abnormalities in the *COL4A5* gene have a significant influence on the rate of progression to ESRD in males with X-linked AS (30, 37). Female carriers of XLAS syndrome are known to experience milder disease than affected males, and X-chromosome inactivation may be a major determinant of the difference in phenotype (36, 38). All affected males and around 95% of females experience microscopic hematuria from early childhood and episodes of macroscopic hematuria may present episodically throughout life (30, 36). In a large cohort study of families with X-linked AS, the type of genetic mutation determined the risk of progression to ESRD and hearing loss (30). Male patients with large deletions, nonsense or small mutations carried a significant 90% probability of developing ESRD, whereas individuals with a missense or a splice mutation had a 50–70% risk of this complication.

In AS, there is a defect in the switch from the α1α1α2(IV) to the α3α4α5(IV) networks during glomerulogenesis and instead the α1α1α2(IV) network predominates in the mature GBM (25, 39). Changes in type IV collagen networks may influence signaling *via* discoidin domain receptor 1 (DDR1) and integrin α2β1 (40, 41), which may in turn alter podocyte function. These extracellular receptors, DDR1 and DDR2, belong to a family of receptor tyrosine kinases, which are important for cellular regulation (42). Receptor tyrosine kinases are typically activated by peptide-like growth factors; however, activation of DDRs is mediated by collagens, which act as DDR ligands (43–45). Mice deficient in α2 integrin do not have a renal phenotype; however, DDR1-knockout mice display thickening of the GBM, podocyte foot effacement, and proteinuria (46, 47). Mice with AS and DDR1 deficiency or haploinsufficiency had improved renal function and increased survival (48). These models suggest a potential role for DDR1 signaling in the pathogenesis of AS; however, exact mechanisms are not fully understood.

The structural components of the glomerulus including the capillary loops, mesangial cells, and matrix and podocytes may also undergo damage due to high-mechanical forces exerting biomechanical strain on glomerular cells. The effects of increased blood pressure were seen in Alport mice treated with l-NAME, which is an inhibitor of nitric oxide synthase and causes hypertension. These mice developed proteinuria and severe ultrastructural defects in the GBM (49). In contrast, the angiotensin-converting enzyme (ACE) inhibitor ramipril delayed the progression to proteinuria in the *COL4A3* knockout mice and has been shown to improve survival in both mouse models and human disease (50, 51). Recent work highlighted that biomechanical strain-sensitive activation of mesangial cell actin dynamics may be important in the pathogenesis of AS. This was demonstrated by the upregulation of endothelin A receptor expression in the glomeruli of Alport mouse models, which lead the formation of mesangial filopodia and the deposition laminin α2 (52, 53) in the GBM. The accumulation of mesangial proteins within the GBM led to the activation of focal adhesion kinase in podocytes and nuclear translocation of NF-κB, which induces a pro-inflammatory state and the release of cytokines, chemokines, and metalloproteinases (54). Interestingly, matrix metalloproteinases not only show increased expression in the glomeruli of Alport mouse models but pharmacological inhibition of these inflammatory mediators before the onset of proteinuria prevented disease progression and improved survival (55, 56). The inhibition of transforming growth factor-β1 was thought to slow disease progression and although this inhibitor prevented GBM thickening, it did not prevent foot process effacement (57, 58). Advances in GBM biology have enabled better understanding of pathogenic mechanisms in AS; however, more research is required to enable the development of therapeutic targets that may delay or stop the progression of the disease.

##### Treatments

Medical treatment using ACE inhibition has demonstrated significant benefits including the reduction of proteinuria and delay of ESRD in AS (51, 59); however, mechanisms of action of these drugs are not fully understood. In a retrospective study, the use of ACE inhibitors appeared to delay renal failure and improve life expectancy in three generations of Alport families (51). Findings resulting from the EARLY PRO-TECT Alport trial of the effects of RAS blockade on the progression of ESRD will be important in supporting the development of emerging candidate therapies (60, 61). At the point of requiring renal replacement therapy, renal transplantation is associated with good long-term outcomes (62). Appropriate donor selection is crucial as the risk of penetrance of *COL4A5* gene mutation is particular high within families, although transplantation among relatives has been reported (63, 64). This method is by no means suboptimal and important points need to be considered, which include the acceptance of donors without proteinuria and the counseling of families who may experience an increased risk of ESRD in the recipient (64).

A small proportion of around 3% of patients with AS suffer from severe post-transplant anti-GBM disease (63). This complication typically presents with hematuria, which progresses to graft rejection. The prognosis for survival in these patients who undergo subsequent transplantation is poor (65). The risk of post-transplant anti-GBM nephritis in patients with AS is increased if the underlying defect is due to a gene deletion (66). Antibodies in anti-GBM are generated against the α5(IV) and α3(IV) chains in patients with X-linked and autosomal recessive AS, respectively (67). Affected individuals display histological similarities to Goodpasture disease; however, post-transplant anti-GBM disease is different in that it does not occur spontaneously and it involves an alloimmune response against normal type IV collagen in the form of foreign antigenic epitopes.

Future alternative options to conventional therapy include gene replacement and several approaches have been employed, which may have broader implications for the development of therapeutic targets that aim to repair the GBM after pathological insult. The insertion of a full-length *COL4A3* under control of a tetracycline inducible promoter into *Col4a3^−/−^* mice significantly reduced proteinuria, increased survival, and restored missing collagen IV networks despite the persistence of ultrastructural damage within the GBM (68). Advances in stem cell technology have also explored the possibility of restoring type IV collagen network to the GBM in Alport AS. Replacement through the transplantation of wild-type bone marrow-derived stem cells into irradiated *Col4a3^−/−^* mice resulted in possible recruitment of podocytes and mesangial cells within damaged glomeruli and a partial re-expression of α3(IV) collagen chains (69). Histologically, these mice had improved glomerular architecture associated with a significant proteinuria compared with untreated *Col4a3^−/−^* mice or irradiated *Col4a3^−/−^* mice that had received bone marrow from adult *Col4a3^−/−^* mice. Stem cell therapies have highlighted novel approaches for the treatment of AS; however, these methods require further refinement before their application in human disease.

#### Heterozygous Mutations in *COL4A3* and *COL4A4*

##### Clinical Features

Individuals with a single mutation in *COL4A3* or *COL4A4* present with microscopic hematuria in childhood and this is due to the diffuse thinning of the GBM. Patients with thin GBMs may have episodic macroscopic hematuria throughout life and are at risk of developing proteinuria and progressive CKD. Approximately 1% of the population is affected by thin GBMs and at least two-thirds have another affected relative (70). These patients do not usually suffer from extrarenal manifestations such as hypertension or deteriorating renal function requiring dialysis or kidney transplantation.

##### Etiology and Pathogenesis

Heterozygous mutations in the *COL4A3* and *COL4A4* gene account for up to 40% of cases in families with thin GBMs and these mutations demonstrate linkage to the *COL4A3/COL4A4* locus, which is also affected in autosomal recessive AS (71, 72). The majority of these patients have non-progressive hematuria; however, a number of individuals will experience a deterioration in renal function with pathological changes, which include GBM thickening, proteinuria, nephrotic syndrome, and progressive CKD. Of the 82 patients from a cohort of patients with thin GBMs who had heterozygous mutations in *COL4A3* and *COL4A4*, around 37.8% developed CKD and 19.5% progressed to ESRD (73).

Patients with thin GBMs rarely require treatment, however, given the variation in presentation in a minority of individuals, long-term monitoring including blood pressure, proteinuria, and renal function is strongly recommended.

#### HANAC Syndrome

##### Clinical Features

Hereditary angiopathy, nephropathy, aneurysm, and cramps comprise HANAC syndrome. The disease phenotype in families is characterized by muscle cramps, mild cerebral small-vessel disease, retinal artery tortuosity, intracranial aneurysms, and renal disease associated with multicystic kidneys, occasional hematuria, and decreased glomerular filtration rate in older patients (74–76). Intrarenal structural abnormalities occur within Bowman’s capsule, tubular basement membrane (TBM), and peritubular capillaries; however, the integrity of the GBM appears normal.

##### Etiology and Pathogenesis

Mutations in *COL4A1* affecting the α1α1α2(IV) network were first identified in families with porencephaly, a condition characterized by cystic and cerebral white matter lesions (77). Small-vessel disease affecting the brain and eye has also been described in a single family with *COL4A1* mutation (78). Missense mutations in the *COL4A1* gene, localized in exons 24 and 25, which affect glycine residues and interrupt the Gly-Xaa-Yaa amino acid repeat have been described in three families with autosomal dominant inheritance of the disease (79). In mice with the *Col4a1* G498V mutation used to model HANAC syndrome, there was evidence of delayed glomerulogenesis and podocyte differentiation without a reduction in nephron number (80). Given the extensive expression of the α1α1α2(IV) network as well as its importance in glomerular development and podocyte differentiation, it is not surprising that mutations in the *COL4A1* gene may lead to a systemic phenotype. Treatment for this condition is largely supportive, and distinct therapeutic targets have not yet been defined.

### Laminin

**Figure 4:**
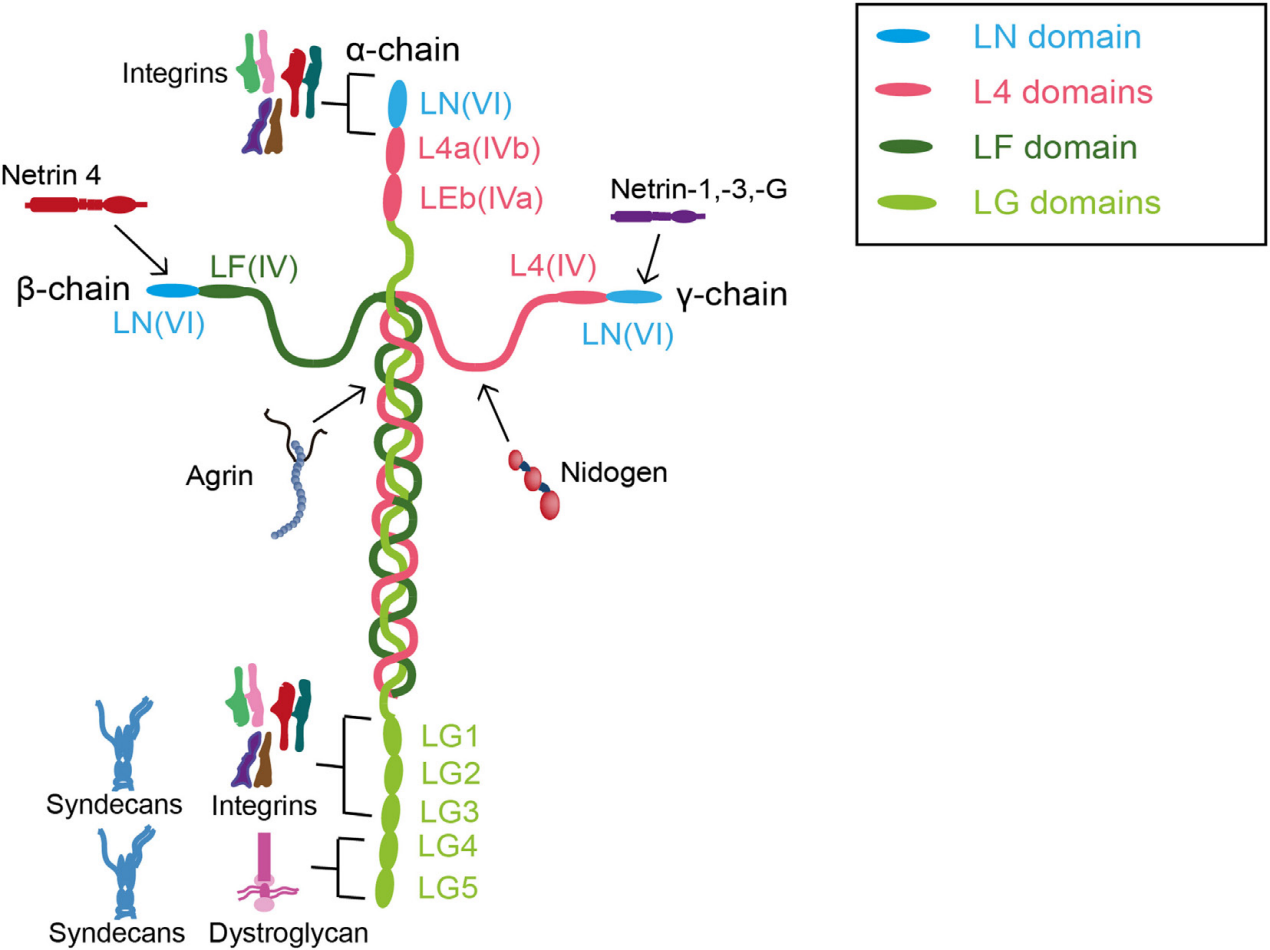
Interactions between laminin and other extracellular matrix components in the glomerular basement membrane. Abbreviations: LF, globular F terminal domain; LG, globular C terminal domain; LN, globular N terminal domain.

Laminins are large glycoproteins that produce α, β, and γ heterotrimers, which provide a structure for the attachment of matrix proteins within the ECM (81). In humans, there are at least 15 distinct laminin heterotrimers composed of an assembly of 4 α-, 4 β-, and 3 γ-chains (Figure 4) (82, 83). Laminin heterotrimers have tissue-specific expression depending on their αβγ composition. The predominant laminin heterotrimer secreted by endothelial cells and podocytes is α5β2γ1 or LM-521, which features in the mature GBM and replaces α1β1γ1 (LM-111) and α5β1γ1 (LM-511), which are present during glomerulogenesis (3, 84, 85). The majority of laminins form cross-shaped structures composed of one “long arm” formed by αβγ chains *via* coiled–coil interactions and disulfide bonding, and three “short arms” with NH_2_-terminal globular (LN) domains (86, 87). These LN domains in the short arms of cross-shaped structures mediate trimer polymerization, which has a significant role in basement membrane formation (88). Another unique feature of laminin is the α-chains that have C-terminal laminin globular (LG) domains at the distal end of the long arm, which binds the laminin trimer to laminin receptors (89).

The LG domain mediates interactions with cell surface receptors including integrins and dystroglycan (90, 91). Integrin α3β1 binds to LM-511 and LM-521 through the LG domain of the α5 chain (26). This integrin is a key surface receptor responsible for capillary loop formation and mesangial cell organization, and podocyte-specific deletion affects normal glomerular development (92, 93). Changes in expression of the α-dystroglycan subunit that binds to laminin occurs in glomerular disease; however, deletion in podocytes did not affect susceptibility to injury or recovery from damage in the kidneys (94–98). In addition, glycosylation of α-dystroglycan which disrupts its binding with laminin resulted in mild podocyte foot process effacement without proteinuria (99).

#### Pierson Syndrome

##### Clinical Features

Pierson syndrome is a rare condition inherited in an autosomal recessive manner with phenotypic variability and characterized by congenital nephrotic syndrome which progresses to ESRD within the first year of life. In some cases, renal manifestations may present as early as the prenatal period with oligohydramios and kidney enlargement, which can be detected using ultrasonography (100, 101). Children with Pierson syndrome are affected by renal impairment soon after birth; however, with renal replacement therapy, some have lived for up to 2 years of age (102). There are currently no reports to date of adult patients with Pierson syndrome. Extrarenal manifestations include fixed pupil constriction (microcoria) but not all patients with Pierson syndrome have eye abnormalities (103, 104). Congenital muscle hypotonia and neurodevelopmental abnormalities may also present in some cases (105).

##### Etiology and Pathogenesis

Pierson syndrome is caused by the homozygous or compound heterozygous mutations in β2-laminin encoded by the *LAMB2* gene (106). Given the variable expression resulting from mutations in the *LAMB2* gene, there appears to be a broad spectrum of phenotypic presentations and screening of this gene is to be considered should there be no mutations found in *NPHS1, NPHS2*, or *WT1* (104). Patients with milder missense *LAMB2* mutations, including R246Q and C321R, present with a less severe renal and extrarenal phenotypes (16, 104). In Pierson syndrome, there appears to be a deficiency of the major laminin protomer LM-521 containing β2-laminin expression, which predominates in mature GBM. Mice with a null mutation in *LAMB2* which display the clinical features seen in Pierson syndrome are a good model for the study of this condition (107). The loss of LM-521 in the normal mature GBM is replaced by LM-511, which usually features in the developmental GBM. The generation of *LAMB2^−/−^* mice has been useful in that they recapitulate Pierson syndrome, and develop congenital albuminuria, podocyte foot process effacement, and are non-viable from 3 weeks of age due to neuromuscular defects and nephrotic syndrome (107, 108). In the *LAMB2^−/−^* mice, there was an ectopic deposition of several laminins, a possible a compensation for the loss of LM-521, and mislocalization of anionic sites despite a structurally normal GBM (109). Like in the human disease, proteinuria preceded structural abnormalities in the podocyte and slit diaphragm in these mice, which suggests the premature onset of pathological mechanisms before the onset of damage within the GBM.

Using the *Lamb2^−/−^* mice in the study of Pierson syndrome has its limitations in that they die at a young age, and therefore the long-term effects on the kidneys and neuromuscular junction are not known. Transgenic mice with varying podocyte expression levels of R246Q-mutant rat β2-laminin were subsequently developed to investigate the degree to which missense mutations in the *LAMB2* gene cause proteinuria. These mice were generated by crossing *LAMB2^−/−^* mice with transgenic mouse lines expressing rat laminin-β2 either in muscle or podocytes (110). In transgenic mice where laminin-β2 was restricted to the kidney, the degree of proteinuria was milder compared with non-transgenic *Lamb2^−/−^* mice, but severe kidney and neuromuscular defects were maintained. Where laminin-β2 was limited to muscle expression, synaptic function was restored; however, mice died from kidney disease at 1 month of age. Transgenic podocyte-specific Lamβ1 expression in *Lamb2^−/−^* mice successfully increased LM-511 trimer deposition, eliminated nephrotic syndrome, and extended survival, highlighting a potential new therapeutic approach (111). These findings implicate potential targets for future therapies and that targeting the kidneys alone may not be effective in Pierson syndrome. Treatment for Pierson syndrome is currently supportive.

### Nidogen

Also known as entactins, nidogen-1 and nidogen-2 are dumbbell-shaped basement membrane proteins with three globular domains connected by two threads separating G-domains (112). Different genes encode nidogen-1 and nidogen-2, and nidogen-1 binds to the short arm of the laminin γ1 chain and type IV collagen, which is thought to bridge these separate networks in the basement membranes (113, 114). It is uncertain if nidogen may have an important role in basement membrane formation, as the deletion of either of the nidogen genes produce viable mice with a normal phenotype (115, 116). Interestingly, nidogen-2 knockout mice are more susceptible to the induction of renal injury compared with the wild-type mice, and these mice develop increased blood pressure, serum creatinine, and albumin excretion (117). Nidogen-2 may have a protective or reparative role in the GBM; however, mechanisms are not known. In mice with simultaneous deletion of both nidogen-1 and nidogen-2, basement membrane formation occurs normally; however, these mice die perinatally and develop subsequent basement membrane abnormalities in the lungs and heart (10). Despite a normal GBM appearance in double nidogen knockout mice, there was phenotypic variability and a subgroup developed renal dysgenesis or agenesis (unilateral or bilateral), hydronephrosis, and polycystic defects. To date, the effect of mutations in nidogen genes in the human kidney is not known; however, quantitative mass spectrometry proteomics have identified that the *NID1* gene, which encodes for nidogen-1, promotes lung metastases of breast cancer and melanoma (118).

### Heparan Sulfate Proteoglycans

The three major HSPGs identified in basement membranes include perlecan, collagen XVIII, and agrin. The commonest HSPGs in basement membranes during development are perlecan and collagen XVIII, whereas agrin predominates in the mature GBM (8, 119, 120). Perlecan binds to nidogen, laminin, and collagen IV through heparan sulfate chains in the immature GBM and after glomerulogenesis, a higher concentration of perlecan is found in the mesangial matrix (121–123). Perlecan mutant mice have normal kidneys; however, they appear to be more susceptible to proteinuria after albumin overload. Mice deficient in collagen XVIII have deranged creatinine levels and display mesangial matrix expansion, but did not display GBM abnormalities (124–126). Recent ultra-high resolution STORM imaging correlated with electron microscopy reveals differences in the distribution of agrin in mouse and human GBM (127). In mice, the C-terminal end of agrin is located adjacent to cell membranes and the N-terminus is found toward the center of the GBM, whereas in humans, agrin is mainly present in the subepithelial area where podocytes reside. Therefore, the phenotype seen in mouse models may not represent directly relevance to human mutations due to interspecies differences in the GBM distribution of HSPGs.

The heparan- and chondroitin-sulfate glycosaminoglycan side chains frequently undergo sulfation, resulting in a negatively charged proteoglycan, which is thought to contribute to charge selectivity within anionic sites in the GBM (9, 128). The anionic sites within the GBM can be detected with polyethyleneimine and cationised ferritin. Agrin has an N-terminal domain bound to the long arm of LM-521 and a C-terminus bound to integrins and dystroglycan, which implicates a potential role in charge selectivity (129). Mice with a podocyte-specific mutation of agrin had fewer negatively charged sites and a shorter length of protein; however, these defects did not result in proteinuria or GBM abnormalities after an albumin overload (9). The deletion of both perlecan and agrin in mice had no effect on permselectivity of the filtration barrier (130). Whether charge selectivity exists within the glomerular filtration barrier is not clear, and these studies confirm that HSPGs may not have a major role in this function.

#### HSPGs in Glomerular Disease

Mutations in HSPGs in human glomerular disease are not well defined and remain limited to immunohistochemical staining in single case reports. Global or segmental loss of staining for heparan sulfate chains in the GBM is evident in lupus nephritis, membranous nephropathy, minimal change disease, and diabetic nephropathy but not in IgA nephropathy or AS (131). The loss of HSPG in the GBM is also observed in individual case reports of C3 glomerulopathy and Denys–Drash syndrome (132, 133). Similarly, animal models including the mouse model of lupus nephritis (MRL/lpr), rat model of active Heymann nephritis, or membranous nephropathy and rat model of streptozotocin-induced diabetic nephropathy show reduced GBM heparan sulfate staining (134–136). A potential mechanism accounting for the reduction of heparan sulfate may be the increase in active heparanase in kidney disease, which shortens these chains through hydrolyzation of glycosidic bonds at specific sites (137). In mouse models of passive Heymann and anti-GBM nephritis, the increase in glomerular heparanase is accompanied by the loss of heparan sulfate, proteinuria, and complement activation. Treatment of these mice with anti-heparanase antibodies reverses proteinuria (138). Decreased expression of heparan sulfate may be due to these domains being masked by the deposition of autoantibodies and immune complexes in the GBM, which is seen in MRL/*lpr* mice and patients with systemic lupus erythematosus (SLE) (139). The reduction in heparan sulfate has been recently linked to complement activation. There appears to be a loss of complement regulator factor H in lupus nephritis and anti-GBM disease, which leads to inadequate complement activation and glomerular injury (140, 141). There is clearly a role for HSPGs in the regulation of the GBM and characterization of physiological networks is required to understand its function in health and disease.

### Fibronectin

Fibronectin is a large 270-kDa glycoprotein with diverse function, which binds to heterodimeric cell surface receptors such as integrins and other ECM components (142). There are three modular domains of fibronectin, which includes type I and II repeats maintained by disulfide bonds and type III repeats characterized by the absence of these bonds that enable it to undergo conformational changes (143–145). Fibronectin can occur in two forms. Plasma fibronectin is secreted into blood from hepatocytes in its soluble form and may integrate into the fibrillar matrix, a role previously attributed to cellular fibronectin (146, 147). Integrins are important in linking fibronectin to the actin cytoskeleton and enabling the process of matrix assembly; however, fibrin organization within fibrils is not known (148). Mechanical forces regulate interactions between fibronectin and collagen; however, these findings have been limited to *in vitro* fibroblasts, and the role of this novel mechanism *in vivo* is not known (149). Fibronectin is not an abundant component of the GBM; indeed, our proteomic analyses demonstrated a fivefold higher abundance of type IV collagen compared with fibronectin in healthy glomerular ECM (11). In disease, however, there appears to be a greater glomerular deposition of fibronectin, and this has been observed in a diabetic nephropathy model (150).

#### Fibronectin in Glomerular Disease

Fibronectin glomerulopathy is inherited in an autosomal dominant manner and is characterized by proteinuria, microscopic hematuria, hypertension, and abnormal renal function. Glomerular deposition of fibronectin can lead to progressive ESRD between the second and sixth decade of life (151). Light microscopy findings include enlarged glomeruli with minimal hypercellularity, and mesangial and subendothelial space deposits. Granular, fibrillary, or mixed deposits within the glomeruli can also be detected by electron microscopy. A significant finding in fibronectin glomerulopathy is the strong positive staining of fibronectin observed in the glomeruli. The immunoreactivity for immunoglobulins, complement proteins, laminin, and type IV collagen was absent or weak. Fibronectin deposition may potentially occur in other glomerular diseases, evident in mouse models of SLE and patients with lupus nephritis (152). However, recent proteomic analyses of laser-captured microdissected glomeruli comparing living-related healthy donors and patients with glomerular disease confirm that fibronectin specifically expressed in patients with fibronectin glomerulopathy (153). Mutations causing fibronectin glomerulopathy are not well studied; however, in a large Italian pedigree, linkage at the *FN1* locus at 2q32 was detected and dominant mutations in this gene contributed to 40% of cases in this group (154).

### Transcription Factors

There are several transcription factors that have a role in regulating the GBM and ECM proteins. The LIM-homeodomain transcription factor encoded by the *LMX1B* gene is highly expressed in podocytes, with LIM domains at the N-terminus facilitating protein interactions and a central homeodomain enabling DNA binding. Mutations in the *LMX1B* gene result in the absence or inactivation of this central homeodomain, which is associated with nail patella syndrome (NPS) (155). In the *LMX1B* conditional knockout mouse, there is a decreased expression of *COL4A3* and *COL4A4* genes in the glomeruli (156). In addition, *LMX1B-*deficient podocytes displayed structural abnormalities including a reduced number of foot processes that were dysplastic and lacked slit diaphragms. The levels of CD2-associated protein and podocin expression were also reduced in *LMX1B-*deficient podocytes, which implicated a potential role for *LMX1B* in regulating these key proteins.

#### Nail Patella Syndrome

Nail patella syndrome, also known as hereditary osteoonychodysplasia, is a rare autosomal dominant disorder presenting with pleiotropic developmental abnormalities of dorsal limb structures involving hypoplasia or absence of the patellae, dystrophic nails, and dysplasia of the elbows and dorsal ilium (157). Phenotypic variability is present despite increased penetrance, and it appears that less than half of affected individuals with NPS develop nephropathy with proteinuria and hematuria (158, 159). Although the majority of patients experience a benign nephropathy, around 30% develop the risk of progression to renal failure.

Patients with NPS have over 140 heterozygous mutations in *LMX1B* with missense, splicing, insertion, deletion, and nonsense alterations (155). *LMX1B* mutations that occur in the LIM-homeodomain are associated with skeletal defects in NPS (160). Mutations affecting the central homeodomain are significantly associated with renal involvement and this pattern appears to cluster in families (161). The microscopic findings of structural renal abnormalities in NPS are fairly non-specific and they include irregular GBM thickening with a “moth-eaten” appearance (162). To date, mechanisms leading to human glomerular disease in NPS have not been characterized and no distinct therapeutic targets have been identified.

### GBM Disease Involving Mutations in Podocyte Cytoskeletal Genes

Myosins contribute to the cytoskeletal machinery of cells, hydrolyzing ATP, and interacting with actin to enable movement along muscle fibers. Non-muscle myosin heavy chain IIA (NMMHC-IIA) is encoded by *MYH9* and is expressed in glomerular podocytes, mesangial cells, and arteriolar and peritubullar capillaries. Mutations in the *MYH9* gene can result in variable phenotypic presentations, which include Epstein and Fechtner syndromes, May–Hegglin anomaly, and Sebastian syndrome. Both Epstein and Fechtner syndromes were once considered to be variants of AS due to similarities in clinical phenotype, which include hereditary nephritis, hearing loss, and ocular abnormalities; however, type IV collagen expression appears to be preserved (163, 164). Patients with Epstein and Fechtner syndromes may also develop thrombocytopenia, macrothrombocytopenia, and leukocyte inclusions (165).

Genotype–phenotype correlations have suggested that mutations in the amino-terminal domain, which binds myosin light chains interacts with actin and utilizes ATP, may cause more severe phenotypic manifestations including nephritis, thrombocytopenia, and deafness before 40 years of age compared with mutations in the carboxy-terminal tail (166). The effects of *MYH9* mutations on the GBM are not known, and microscopic findings have been fairly non-specific and likened to those observed in AS, with focal GBM thinning and foot process effacement (167). The expression of NMMHC-IIA in podocytes is reduced in *MYH9*-related disease (167, 168), but it is unclear whether these alterations affect GBM synthesis and organization. Our current knowledge on the long-term consequences of *MYH9* mutations on the GBM remains limited, and *MYH9* does not appear to be the only contributing factor to the spectra of conditions affecting the podocyte cytoskeleton.

### GBM Disease Caused by Abnormal Regulation of Adhesion Receptors

The GBM is composed of condensed sheets of ECM with a supramolecular assembly built around two major networks of laminin and collagen IV (169). The role of the GBM is to support its ECM components and provide a scaffold for adjacent endothelial cells and podocytes. Cell–matrix interactions are key to the integrity of the filtration barrier maintained by cells adhering to the ECM through adhesion receptors, which include integrins, syndecans, and dystroglycan (13).

Integrins are αβ-heterodimeric cell surface receptors that engage with the ECM to mediate cell–matrix signaling, and the α3β1 heterodimer is particularly important in linking the podocyte and GBM (170). The importance of the α3β1 heterodimer is evident in mice lacking the integrin α3 subunit, which are non-viable within the first day of life due to developmental defects in the kidneys and lungs, accompanied by the loss of specialized morphology and thickened irregular GBMs (93). The podocyte-specific deletion of α3 subunit results in an abnormal renal phenotype manifesting as nephrotic syndrome and subsequent renal failure (171). Individuals homozygous for mutations in the integrin α3 gene, *ITGA3*, have disrupted basement membrane structures and compromised barrier functions. These patients develop congenital nephrotic syndrome, interstitial lung disease, and epidermolysis bullosa (172). Mice with the homozygous deletion of integrin β1 are not viable beyond the embryonic phase (173) and podocyte-specific deletion of the β1 subunit in mice causes early renal failure at 3 weeks of age, progressive podocyte apoptosis and capillary loop and mesangium degeneration (174). Mutations of the integrin β1 subunit in humans have not yet been described.

There is also a significant association between the tetraspanin CD151 and integrins in particular α3β1, which may have a role in cell migration (175). The global and podocyte-specific deletion of *CD151* gene in mice results in proteinuria accompanied by the focal glomerulosclerosis, disorganization of the GBM, and tubular cystic dilation (171). Interestingly, the phenotype of *CD151* knockouts significantly depends on the background of mouse used. *CD151*-deficient mice bred on an FVB background develop spontaneous and severe glomerular disease; however, this phenotype was not recapitulated in C57BL/6 knockout models (176). The *CD151* knockouts bred on a C57BL/6 background only display significant proteinuria when challenged with induced hypertension. Patients with a frameshift mutation in *CD151* develop a similar phenotype to mice, which includes focal thickening and irregularity of the GBM as well as combined hearing loss and skin defects (177).

### Genetic Disorders of the TBM

The TBM lies beneath the tubular epithelial cells and is in continuity with the interstitial connective tissue (178). Currently, there are no known genes exclusively linked to TBM abnormalities; however, these abnormalities are present in genetic disorders affecting the kidneys and often present alongside GBM or other extrarenal basement membrane phenotypes. The GBM is well preserved in HANAC syndrome, which is associated with mutations in the human *COL4A1* gene; however, splitting and thickening of the TBM is observed (79). Defects in laminin α5, a major GBM and TBM protein component encoded by *LAMA5* in mice manifest as GBM abnormalities, incomplete glomerular vascularization and polycystic kidneys, and defective interactions between tubular epithelial cells and the ECM may be the cause of cyst formations (179, 180). In AS, progressive disorganization of the GBM is followed by pro-fibrotic changes in the renal interstitium, which is characterized by abnormalities in the proximal renal tubular epithelium and defects in ECM turnover, leading to deterioration in renal function (49, 181, 182). These few examples demonstrate that abnormalities in the TBM appear to occur alongside GBM defects; however, genetic and pathogenic mechanisms causing disease are yet to be fully understood.

## Summary of Research Advances

There have been significant research advances in glomerular disease since the early 1990s and the discovery of genetic disease associated with GBM defects and nephrotic syndrome. However, there remain unmet needs in the diagnosis, clinical care, and disease management. Importantly, there is currently a lack of targeted treatments across the spectrum of GBM conditions. Given the pivotal role of the GBM particularly in AS, a key area of focus is early diagnosis and treatment of disease, which has significant evidence in improving the long-term prognosis (51). The exponential discoveries through next-generation sequencing has enabled better understanding of the heterogeneity of GBM disease, which allows the integration of conventional therapies with a more stratified approach to treatment. Clinicians will therefore be able to avoid invasive biopsies and adjust tailor medications to a patient’s needs more effectively. In addition, patients and families will have the opportunity to plan based on informed genetic counseling. The discovery of new therapeutics through the collaboration of the patients, clinicians, scientists, and industry is therefore necessary, to treat and even possibly prevent foot process effacement and GBM abnormalities (31). This in turn will generate a personalized diagnostic approach and potential therapeutic targets for the treatment of genetic kidney diseases affecting the basement membrane.

## Patient Perspective

Living with kidney disease presents challenges for families and around 4 in 10 patients have experienced a delay in or lack of diagnosis (183), which may result in inappropriate management. Diseases of the GBM are considered to be rare diseases, which affect the minority of people among other more prevalent conditions in the general population. To date, between 5,000 and 8,000 distinct rare diseases have been reported and in the UK, around 1 in 17 people (approximately 3.5 million) will develop a rare disease at some point in their lives (184). Of the rare diseases, around 75% of children are affected and at least 80% have had a genetic component identified. Although clinical teams can provide their expertise in treating families with rare diseases, it is important to recognize the personal challenges that patients’ encounter, which include pathways to getting an appropriate diagnosis, implementation of care, and patient support networks. In addition, there are international efforts working toward the development of 200 new therapies for rare diseases by 2020 (35).

Research in to rare diseases is urgently required to enable better prognoses and targeted treatments for patients and their families. To support people with rare disease, patients’ advisory groups play an important role in helping families through websites, leaflets, and personal contact. For genetic diseases of the GBM, an example organization is Alport UK, which is a patient-led organization dedicated to empowering people who live with AS through the provision of support and information for this condition (185). Alport UK works with a multi-disciplinary internatonal collaboration consisting of researchers, clinicians, academics, pharmaceutical companies, and other patient groups. This valuable partnership enables families and clinicians to access up-to-date and accurate resources, which aids clinical management toward a better quality of life and understanding of the AS. Another role of patient organizations is to assist with recruitment into national patient registries. These efforts are crucial to improving understanding about the natural history rare diseases, which may also inform the causative pathways and improved management for other more common disorders.

## Summary/Conclusion

Over the last two decades, important advances have been made in understanding the composition and function of the GBM, which is important for maintenance of filtration barrier integrity. Studies of core ECM components including laminin, collagen IV, HSPG, and nidogen in health and disease have highlighted the importance of these basement membrane proteins in enabling the GBM to function as the primary filtration barrier of the kidney. Significant progress in the application of global approaches including proteomics analyses has highlighted novel genotype–phenotype correlations, and thus enabling a broader range of molecular diagnoses for rare or unclassified glomerular diseases. In addition, these innovative approaches will enable the development of stratified therapeutic targets and guide the prognosis counseling of families with GBM diseases.

## Author Contributions

CC and RL research the topic and wrote the review. CC prepared the figures.

## Conflict of Interest Statement

The authors declare that the research was conducted in the absence of any commercial or financial relationships that could be construed as a potential conflict of interest.
